# Parents' WhatsApp coping resources in the context of ongoing political conflicts: An ecological exploration

**DOI:** 10.1002/ajcp.70017

**Published:** 2025-09-12

**Authors:** Daphna Yeshua‐Katz, Stav Shapira, Orna Braun‐Lewensohn

**Affiliations:** ^1^ The Charlotte B. and Jack J. Spitzer Department of Social Work Ben‐Gurion University of the Negev Beer‐Sheva Israel; ^2^ School of Public Health, Faculty of Health Sciences Ben‐Gurion University of the Negev Beer‐Sheva Israel; ^3^ Conflict Management and Resolution Program Ben‐Gurion University of the Negev Beer‐Sheva Israel

**Keywords:** Bronfenbrenner's ecological theory, coping, online support groups, political conflicts, social media, WhatsApp

## Abstract

Mobile technologies have become significant resources for crisis communication and social support in recent years. However, despite empirical evidence pointing to the centrality of these technologies for parenthood in everyday life, it is yet unknown how parents' coping resources play a role in the digital environment. In this study, we examined how parents cope with prolonged political violence online, based on Bronfenbrenner's ecological theory and the three levels of coping it encompasses: personal, family, and community. We employed a photo‐elicitation technique during in‐depth interviews with 21 parents residing in communities near the Israel‐Gaza border, to identify digital coping strategies in WhatsApp groups. The data were collected in January 2023, prior to the outbreak of the October 7th Israel‐Gaza war, and therefore reflect coping processes during a period that alternated between relative calm and episodes of escalation. Theoretically, our findings contribute to expanding the core concepts of coping theories, traditionally studied in offline environments, to the digital realm. Empirically, our analysis revealed that participants developed digital coping methods at two ecological levels: personal and community. At the personal level, our participants used local WhatsApp parent groups (WPGs) to manage stress by exchanging emotional and instrumental support and venting emotions. At the community level, our participants indicated that local WPGs could help address emergencies collectively through the provision of instrumental support, emotional support, relief through humor, and as a platform for venting. Our study, by identifying how they use local digital settings, indicates the ways residents can potentially increase their personal and community resilience.

## Highlights

- Local WhatsApp parent groups serve as critical digital coping resources for parents living under political violence.
- Drawing on ecological and coping theories, we examined parents' digital coping strategies.
- Parents developed coping methods at two ecological levels: personal and community.
- WhatsApp supported personal stress management and collective emergency response.
- These groups functioned as both adaptive and maladaptive coping resources.

## INTRODUCTION

The challenge of living in situations of ongoing political violence exposure—such as war and armed conflict—has received increasing attention from researchers, policymakers, and practitioners worldwide. The adverse mental health effects of living in conflict‐affected areas have been described in an extensive body of literature (Greene et al., 2018). However, additional evidence suggests that most individuals do not develop major mental health morbidities because of political violence exposure (Braun‐Lewensohn & Sagy, 2014; Shapira et al., 2020). Instead, they manifest resilience and effective coping abilities (Braun‐Lewensohn & Mosseri Rubin, 2014). Having said that, recent evidence has identified parents of small children as one group suffering from a dramatic decline in perceived resilience levels during political conflicts (Shapira et al., 2020).

One of the main ways individuals cope with adversities is via their ability to access social support, assuming it exists (Braun‐Lewensohn, 2015). In recent years, mobile technologies have become significant resources for crisis communication and social support (Stephens et al., 2020). Despite growing evidence pointing to the centrality of social media for parenthood in everyday life, it remains underexplored how these platforms shape coping resources and social support systems in times of crisis such as ongoing political violence.

Our research, grounded in social constructionism (Berger & Luckmann, 1989), suggests that social realities evolve through human interaction and cultural narratives, shaping individual and communal responses to adversity. People construct meaning within their cultural context, particularly through technology, which is significant in our daily life (Lupton, 2022, 2023). As Lupton (2022, 2023) argues, technological entities do not exist independently; rather, they are interwoven with sociocultural implications and practices. In this study, we built upon Lupton's work by connecting it to Bronfenbrenner's ecological framework, in which the nested systems influencing individual and collective behaviors. Thus, drawing on Bronfenbrenner's (1992, 1986) ecological theory and various coping theories (Carver et al., 1989; Carver, 1997; Cutrona & Suhr, 1992), we investigated the role of digital coping mechanisms, exemplified in local WhatsApp parent groups (hereafter, WPGs), in response to prolonged political violence. Specifically, we investigated how parents frame and perceive the types of coping resources offered in Israeli WPGs during times of increased violence and relative calm, as well as the potential drawbacks and adverse effects of using online groups on mobile media in such a context. Guided by Lupton's (2022, 2023) social construction of technology framework, we explored how parents actively shape and redefine the use of WPGs to address their unique coping needs.

Children are particularly vulnerable in conflict zones (Cummings et al., 2017; Tol et al., 2013). Studying how parents cope can shed light on protective factors that support children's emotional and psychological development (Sousa et al., 2025; Vostanis, 2021). As these groups can provide a platform for various types of social support and can help mitigate the psychological impact of living in conflict zones on families and communities, understanding the role of digital groups can inform strategies to strengthen community resilience against the stress of living in such areas. Moreover, insights from our study can guide policymakers in developing support systems that leverage technologies for community support and lead to better interventions aimed at aiding those living in conflict zones.

### Ecological systems theory

Bronfenbrenner (1986, 1992) posited in his ecological theory of human development that individuals exist as members within a larger context characterized by nested systems. These systems, instrumental in supporting social development, range from the immediate family system, where individual members interact and grow, to the broader community system, which “holds” the family. Each system provides a myriad of options and opportunities for growth, contributing to an ever‐expanding diversity of experiences. Ecological systems theory presents a valuable perspective for understanding and exploring the resources people utilize to cope with life's challenges. In the case of coping with stressors, access to these “systems” enables individuals to have more social knowledge, find social support, and have an increased set of possibilities to manage adversities. More importantly, access to family‐level and community‐level coping resources has a strong influence on personal‐level coping resources. Such access increases individuals' accurate appraisal of a stressful situation, as well as the appraisal of available coping strategies (Afifi et al., 2020; Long, 1998; Shapira et al., 2025). Coping with stressors is thus explored as a process that occurs within and across these three levels: (1) individual, (2) family, and (3) community (Bronfenbrenner, 1986; Bronfenbrenner, 1992; Elfassi et al., 2016). In this study, *community* is conceptualized as the intersection of three overlapping spheres: (a) a shared geographic locale (Gaza‐envelope municipalities), (b) the offline social network of parents bound by their children's schooling, and (c) the *digital* space created by their WPG. This hybrid definition aligns with Bronfenbrenner's mesosystem (linkages among immediate settings) and exosystem (structures that indirectly shape daily life), while foregrounding Lupton's claim that technologies are themselves sociocultural sites. It is this tri‐layered, digitally mediated collectivity that we analyze when we speak of *community‐level* coping.

#### Individual coping resources

This term refers to individuals' capacity to identify and utilize both internal and external resources, such as social support, to foster their health and well‐being (Eriksson, 2017), and includes the ability to recognize the various resources available to individuals and effectively leverage them in a manner that promotes their physical and psychological welfare. At the individual level, seeking social support can positively impact people's well‐being and their approach to parenting during continuous conflicts. Social support acts as a protective barrier, mitigating the impact of stressors on emotional responses (Lazarus & Folkman, 1984). An additional protective factor at the individual level is Antonovsky's (1987) sense of coherence—a cognitive orientation whereby one views the world as comprehensible, manageable, and meaningful. A strong sense of coherence acts as a protective factor, leading to lower threat perceptions in the context of war (Braun‐Lewensohn et al., 2011; Sagy & Braun‐Lewensohn, 2009). Conversely, a weak sense of coherence may lead to heightened stress responses and maladaptive coping (Hogh & Mikkelsen, 2005), leading to psychological problems and stress responses (Bar et al., 2025; Eriksson, 2017).

#### Family coping resources

Living in a conflict‐affected area may jeopardize parents' protective role as they routinely experience threats to their ability to care for their children, potentially resulting in profound distress (Sousa & El‐Zuhairi, 2017). The resilience of the family system can be defined as its capacity to endure or recover from any adversity that threatens its stability or development (DeHaan et al., 2013; Masten 2018; Walsh, 2016). Walsh (2016) used the term “family shock absorbers” to describe this phenomenon. Coping with a short‐term crisis is not the same as coping with the kind of cumulative strain that emerges from living in conflict‐affected areas. Some families may cope well with the former but are overwhelmed by the strain of the latter (Walsh, 2016). Family‐level impacts are defined as interactions or mechanisms that directly strengthen the family unit, such as joint problem‐solving, collaborative coping strategies, or shared emotion regulation that involve multiple family members working together (Neal & Neal, 2013; Paat, 2013).

Social support at the ecological family level significantly impacts parental stress and well‐being, as evidenced by various studies. Social support, both received and provided, plays a crucial role in mitigating stress and enhancing life satisfaction among parents, particularly those facing challenging circumstances such as having a child with cancer (Melguizo‐Garín et al., 2022). The presence of social support is directly linked to reduced parental stress, as seen in first‐time mothers, where higher social support correlates with lower stress levels (Hutagaol & Rahayu, 2023). Furthermore, social support has been shown to have a protective effect against economic pressures and depressive symptoms, which are significant contributors to parenting stress, especially in low‐income families (Negi and Sattler 2025). In the context of parenting programs, different dimensions of social support, such as formal and informal support, influence parental outcomes, including stress and child‐rearing attitudes, highlighting the importance of tailored support interventions (Álvarez et al., 2021). Additionally, family structure and young adults' perceived social support also affect their life satisfaction and psychological distress, suggesting that a supportive family environment can serve as a protective factor against stress (Almeida and Araujo 2024). This association further highlights the importance of understanding and enhancing parental coping resources for the entire family unit (Patterson, 2004).

#### Community coping resources

It has increasingly been recognized in the literature that individuals cope collectively in social contexts, with stressors affecting individuals and their social networks, and social networks influencing how individuals cope with stress (Dekel & Nuttman‐Shwartz, 2009; Helgeson et al., 2018; Kimhi et al., 2020). Coping as a collaborative process becomes even more relevant when exploring collective crises such as armed conflicts, which affect entire communities. Community resilience, similar to family resilience, but on the part of a whole community, has been documented as a key factor in the process of community coping with political violence (Braun‐Lewensohn & Sagy, 2014; Shapira, 2022; Zelis & Shapira, 2025). This resilience includes community preparedness, social support, trust, collective efficacy, place attachment, and strong leadership, all assets in times of threat (Shapira, 2022).

In times of crisis, communities gather and make use of all their resources for the common good. Once the crisis is over and everyone returns to routine, this type of coping may erode (Braun‐Lewensohn, 2014). Studies investigating the interplay of personal and collective resources have indicated that community coping resources have a slight positive effect on personal resources, which, in turn, take over and become the most valuable resources in coping with recent life events (Anson et al., 1993; Bar et al., 2025; Muhonen & Torkelson, 2008). When stress affects social functioning, personal resources facilitate the mobilization of whatever collective resources are available (Anson et al., 1993; Bar et al., 2025).

Research indicates that parents suffering from political violence rely on both informal and formal support systems at the community level (Sousa & El‐Zuhairi, 2017). *Informal support* arises naturally from personal relationships and community ties, fostering trust, understanding, and reciprocity. It helps build resilience, as friends, family, and neighbors provide comfort, advice, or practical help during difficult times (Gill et al., 2023). *Formal support*, offered through community‐based programs, provides structured resources, counseling, and organized activities tailored to parents' needs. These programs, along with community‐based children's groups, enhance problem‐solving by the sharing of resources and information (Owen & Anderson, 2017).

Although Bronfenbrenner's theory was fully developed by the turn of the 20th century (Rosa & Tudge, 2013), the influence of growing up in the digital era could not, of course, have been taken into account at the time. As digital media, in particular smartphones, have become ubiquitous, and as most aspects of our lives now take place on social media (Deuze, 2023), we would argue that it is time to study, through the lens of ecological theory, the role of digital media in the way we cope with life's adversities. In the current digital age, social media group applications (e.g., WhatsApp and Facebook groups) often complement real‐life collective circles and constitute a primary online connection for most individuals. As Lupton (2022, 2023) contends, technological entities do not operate in isolation; rather, they are saturated with sociocultural significance and practices. In the context of social media platforms, users and communities actively reshape these digital environments into arenas for emotional, informational, and networking assistance, thereby illustrating how such digital instruments are socially formulated to mirror and address societal issues.

### Digital coping with adversities

Integrating Bronfenbrenner's ecological theory with the realities of the digital era offers a new perspective on coping mechanisms in the face of adversity. Carver et al. (1989) identified 14 scales of coping, ranging from active/planning strategies to maladaptive behaviors such as denial and substance use (Carver, 1997, 1989). These scales are particularly relevant when considering the influence of digital media in our daily lives. Cutrona and Suhr's (1992) social support behavior code (SSBC) provides a framework for understanding the various forms of support that can be obtained through digital means. Informational support includes the sharing of advice, suggestions, and information to help solve problems. Emotional support involves the offering of empathy, love, trust, and care, which are vital for reducing feelings of anxiety and stress. Esteem support focuses on affirming a person's skills and qualities, thus boosting their self‐confidence and self‐esteem. Network support gives individuals a sense of belonging by connecting them with a community that shares similar interests and concerns, reinforcing their social ties. Lastly, tangible assistance, or instrumental support, includes any material or financial aid provided to someone in need. Together, these support mechanisms are integral to helping individuals navigate the challenges they face, particularly in the digital environment where traditional forms of physical support may not be as readily available.

This spectrum of support aligns with Carver's (1989) scales, situating them within the contemporary context where social media and other digital tools are prominent. The advent of smartphones and social media platforms has expanded the avenues through which individuals seek and receive support, paralleling Carver's coping strategies within a digital framework. These platforms have proven invaluable in crises, such as the 2022 Russian invasion of Ukraine, when nongovernmental organizations and volunteers used social media to provide relief and disseminate critical information (Byrska, 2022). Social media not only offers a rapid exchange of information and network support but also a space for communal coping, enhancing personal and collective resilience (Neubaum et al., 2014; Shaw et al., 2013). However, the pervasive influence of digital technologies on coping is not without its drawbacks. Issues such as misinformation, emotional distress, and social overload point to the potential negative consequences of digital coping, reflecting Carver's dysfunctional coping scales, such as denial and self‐distraction, highlighting the complex interplay between coping strategies and digital consumption (Bradshaw & Howard, 2019; Drouin et al., 2020; Hampton et al., 2016). These complexities require a nuanced exploration, particularly as they pertain to the domain of parental coping with ongoing political violence.

### Parents' digital coping in the context of political violence

Parent‐to‐parent peer support in digital environments, particularly via social media, can create a safe space to collectively share experiences, exchange support, and acquire information, fostering personal growth and meaningful relationships. In turn, WPG membership can influence parents' mental health and well‐being (Roitman & Yeshua‐Katz, 2022). Compared to the wealth of research on children and adolescents in the context of political violence (Lemish & Götz, 2014), there have been fewer studies focusing on the digital coping of adults, and even fewer on the specific coping of parents of dependent children. The sparse evidence that is available underscores the added distress experienced by such mothers and fathers due to the difficulty they have in protecting their children from the physical and mental consequences of hostilities, as well as concerns regarding the children's safety (Sousa & El‐Zuhairi, 2017). WhatsApp parent groups have been identified as a shared resource of emotion‐ and problem‐focused support for coping with political violence (Roitman & Yeshua‐Katz, 2022). However, as alluded to above, there are also downsides to such social media usage. Research has indicated that active social media use (e.g., posting on social media groups) during a crisis is associated with increased parental anxiety (Drouin et al., 2020). Thus, considering the potential for social media to have both negative and positive impacts, it is essential to identify factors related to social media use that are involved in the process of parental adjustment for successful coping with ongoing political violence.

By applying Lupton's (2022, 2023) concept of the social construction of technology within Bronfenbrenner's ecological framework, this study highlights how parents individually and collectively reconfigure WhatsApp groups to serve as adaptive coping mechanisms. Doing so underscores the profound interplay between technology and culture, where digital platforms are not just passive tools but dynamic spaces shaped and reshaped by individuals to express their agency, meet collective needs, and foster resilience in communities navigating political violence.

### The groups under study

The current study focuses on the Israeli communities adjacent to the Gaza Strip, often referred to as the “Gaza envelope.” This area is one of the most volatile regions in Israel. Parents in this region live under the constant threat of rocket attacks, with alarms sounding frequently and providing only seconds to find shelter (Paryente & Kalush, 2020). This precarious reality shapes their daily lives, as families must navigate the psychological toll of chronic insecurity while maintaining routines for their children. Research has shown that prolonged exposure to such conflict increases rates of anxiety, posttraumatic stress disorder, and other stress‐related symptoms among both parents and children (Dekel & Nuttman‐Shwartz, 2009; Greene et al., 2018). The data for this study were collected in January 2023, prior to the outbreak of the October 7 2023 Israel Gaza war, and therefore reflect coping processes during a span marked by intermittent calm and recurrent escalations. The ecological macrosystem influences this reality in several ways. Cultural narratives in Israeli society emphasize resilience and solidarity, which encourage communities to band together in the face of adversity but may also place pressure on individuals to suppress emotional vulnerabilities (Levi‐Belz et al., 2025). These lived experiences are situated within an ongoing conflict that further exacerbates structural inequities. The communities in the Gaza envelope have borne the brunt of conflict‐induced disturbances more than any other area in Israel. Threats include the launching of rockets or setting off of incendiary balloons/kites by Hamas (the governing authority of the Gaza Strip), as well as terrorists' infiltration, leading to loss of life, significant property and economic damage, and consequent psychological and behavioral consequences (Gelkopf et al., 2012a; Stein et al., 2018).

Within this context, WPGs function as critical tools for coping and communication. WhatsApp, a widely used instant messaging app with over a billion users globally, has been crucial during emergencies and natural disasters. It offers various multimedia functions such as voice and video calls, as well as image, audio, and video message sharing. Its ease of use and cost‐effectiveness (only requiring an internet data plan) contribute to its popularity. During the 2014 Israel‐Gaza conflict, WhatsApp was pivotal for Israelis in staying informed and connected, enhancing community and national unity amidst political violence (Malka et al., 2015). It served as a key communication channel for news and checking on relatives, especially for those near war zones, and helped users feel calmer.

WhatsApp, which has achieved widespread popularity in Israel, does not require advanced digital literacy for its effective utilization. According to a recent digital media use annual report (2023), an impressive 90% of Israeli adults are users of WhatsApp, and a significant proportion (76%) express a reluctance to discontinue its use. Additionally, studies from the fields of telemedicine and education in Israel have posited that WhatsApp serves as a facilitative tool in narrowing the divide between the more central and more peripheral regions of the country. Such research consistently reveals an absence of marked disparities in the dialogs across varying socioeconomic strata (Addi‐Raccah & Yemini, 2018; Barayev et al., 2021).

As far as we know, comprehensive academic studies on Gazan parents' and caregivers' use of WhatsApp groups are nonexistent. Our study focuses on Israeli parents and caregivers near the Israel‐Gaza border (i.e., rather than on Gazan parents and caregivers in Gaza) due to practical constraints, including significant logistical and security challenges that restrict Israeli scholars' access to Gaza. These limitations influenced our decision to examine how Israeli families use WhatsApp groups to cope with chronic political violence. The unique circumstances in this region—frequent rocket attacks, protective shelters, and a history of conflict—create a distinct digital communication dynamic. Additionally, access to participants and shared cultural and linguistic contexts on the Israeli side enabled us to gather reliable data. Although our focus provides valuable insights, we acknowledge the need for complementary research in Gaza to offer a fuller understanding of the region's challenges.

Adopting a social constructionist lens, our research aim was to examine how parents framed and interpreted their coping strategies in digital spheres, notably within local WPGs, in the shadow of enduring political unrest. This exploration was grounded in Bronfenbrenner's ecological framework, emphasizing three levels of coping: individual, familial, and communal.

## METHODS

### Design and approach

Informed by the social construction perspective, we aimed to delve deeper into understanding how parents structured and perceived their coping mechanisms in digital realms. A key methodological choice in our study was the use of the photo‐elicitation method, which aligns closely with the constructivist epistemological paradigm (Harper, 2002; Hutchinson, 2016). This method involves incorporating participant‐generated visual artifacts—such as screenshots from WPGs—to co‐construct meaning and deepen understanding of the phenomenon under study. Photo‐elicitation engages participants in the research process by allowing them to select and interpret visual data that reflect their lived experiences, fostering a collaborative and participant‐centered approach to knowledge generation. By grounding the interviews in these visual artifacts, we enabled participants to narrate their experiences on their own terms, enhancing the richness and authenticity of the data while emphasizing their role as co‐creators of meaning.

### Participants & sampling

Data were collected January–May 2023, a span that alternated between calm and escalation, enabling participants to describe both realities. We recruited 21 parents (Table 1) through purposive maximum‐variation sampling. Invitations were sent via (1) municipal mailing lists, (2) resilience‐center lists, (3) PTO WhatsApp groups, and (4) snowball referrals. A brief screening recorded demographics, place of residence (kibbutz/moshav, town ≤ 20,000, city > 20,000), and each parent's WPGs. To maximize structural diversity, we drew members from four WPG archetypes: classroom (*n* = 8), PTO (*n* = 6), neighborhood safety/resilience (*n* = 4), and mixed‐interest community (*n* = 3). Although every group was parent‐run, they differed in size (40–300 members), posting rules (announcement‐only vs. open chat) and age (1–7 years). Such heterogeneity is important because normative tone, information flow, and gate‐keeping vary by WPG type and shape coping (Addi‐Raccah & Yemini, 2018). Following Ussher et al. (2008), we included both active and former members to capture factors influencing participation and retention. WPGs offered a locus for information, support, and collective action, yet remained one layer within broader community and institutional dynamics.

**Table 1:** Participants' information.

Name	Type of locality	Age	Gender	Education level	Country of birth	Marital status	No. children	Income
Gila	Urban	42	Female	MA	Israel	Married	3	A
Yoav	Urban	44	Male	BA	Israel	Married	3	A
Amit	Rural	39	Female	High school	Israel	Married	3	L
Yafit	Rural	40	Female	MA	Israel	Married	3	H
Roy	Rural	38	Male	MA	Israel	Married	3	L
Esther	Rural	33	Female	MA	U.S.	Married	3	H
Ye'ela	Rural	45	Female	BA	Israel	Divorced	2	A
Ella	Urban	39	Female	BA	Israel	Married	2	H
Ruby	Urban	41	Male	BA	Israel	Married	2	H
Yevgeny	Urban	41	Male	MA	Ukraine	Married	3	H
Rachel	Urban	36	Female	BA	Former Soviet Union	Married	3	L
Natalie	Rural	36	Female	BA	Israel	Married	4	L
Irit	Rural	44	Female	BA	Israel	Divorced	3	A
Orit	Rural	39	Female	BA	Israel	Married	2	L
Ruth	Rural	43	Female	BA	Former Soviet Union	Married	2	H
Hemda	Rural	44	Female	High school	Israel	Married	5	A
Yaron	Rural	30	Male	BA	Israel	Married	2	Lo
David	Urban	37	Male	High school	Israel	Married	2	A
Diana	Urban	35	Female	BA	Israel	Married	2	L
Yael	Urban	39	Female	MA	Israel	Married	3	A
Inbal	Urban	37	Female	MA	Israel	Married	4	H

Abbreviations: A = average income, Income = income level, H = higher than average, L = lower than average.

### Procedure

A team of 12 undergraduate students, mentored by the first author, conducted face‐to‐face, phone, or Zoom interviews that lasted between 40 and 90 min and were audio‐recorded and transcribed. During the interviews, research team members used the screenshots that had been provided by the participants to stimulate conversation and provide insight into the role of communication in the groups based on the ecological approach's different levels. The objective of the interviews (see interview protocol in Appendix A) was to gain a clearer understanding of how participants perceived their coping experiences within their local WPGs amidst ongoing conflict. This understanding spans three levels as outlined by the ecological theory: personal, family, and community (Bronfenbrenner, 1986; Bronfenbrenner, 1992).

### Ethical considerations

Before data collection, the study was approved by the Ben‐Gurion Department of Social Work Ethics Committee. Participants gave written consent for audio recording, screenshot sharing, and publication of paraphrased excerpts. Screenshots were deidentified (cropping names/photos, redacting numbers) and stored on an encrypted server; published excerpts were translated, paraphrased, or composite‐blurred to prevent traceability.

### Data analysis

The research corpus included 21 interview transcripts analyzed using Atlas.ti software. A hybrid analytical process combined theory‐driven and data‐driven interpretations to ensure a robust and nuanced understanding of the data. To enhance credibility and trustworthiness, we employed a multi‐step process of credibility checks alongside a rigorous analytical approach. Initially, trained undergraduate students who collected the data performed the first round of coding, leveraging their close engagement with both the interview content and the visual data (e.g., screenshots and participants' interpretations shared during the photo‐elicitation process). Their initial context‐sensitive interpretation served as the foundation for further analysis. Each author then independently coded the data, incorporating insights from both visual artifacts and interview transcripts to ensure a comprehensive analysis. Subsequently, the authors collaboratively compared their analyses with the students' initial coding (Burawoy, 2009), identifying areas of alignment and divergence. In cases of disagreement, the authors engaged in in‐depth discussions until consensus was achieved. This iterative process ensured that the final themes reflected the complexity and richness of the data while remaining grounded in the participants' lived realities.

The analytical framework was guided by Bronfenbrenner's ecological systems theory (1992, 1986) and coping strategies frameworks (Carver et al., 1989; Carver, 1997; Cutrona & Suhr, 1992). These frameworks informed the formulation of research questions, the review of relevant literature, and the creation of a thematic codebook tailored to digital coping strategies at each ecological level. The open coding process allowed for the integration of theory‐driven codes while remaining flexible enough to incorporate new, emergent codes based on participants' narratives. This iterative approach led to the development of digital coping strategy‐specific codes. For example, Carver's (1997, 1989) brief‐COPE scale and Cutrona and Suhr's (1992) SSBC framework were used to identify participants' efficient and inefficient coping strategies, such as positive reframing, humor, and emotional support, within the context of their local WPGs. These frameworks helped us deductively code instances where participants employed the WPG to cope with distress and uncertainty. In addition, inductive analysis revealed unexpected coping strategies. For instance, some participants disengaged from the WPG to manage stress (individual behavioral disengagement), whereas others used information shared in the WPG to plan emergency actions (individual planning). Participants also utilized the WPG to organize collective efforts, such as coordinating evacuations by broadcasting videos from evacuation locations (collective instrumental support). These findings demonstrate the integration of theory with emergent data to provide a comprehensive understanding of digital coping strategies.

During axial coding, the team agreed on three operational tests: unit of action (who instigates the behavior), intended beneficiary (who is meant to gain), and degree of joint coordination. A code was marked *family* when at least two household members enacted or jointly benefited from the behavior (e.g., parents deciding *together* to move children into the shelter). When the action involved multiple households or explicit concern for neighborhood welfare, it was marked *community*. Behaviors confined to the individual actor—even if later shared—remained *personal*. These rules guided the relocation of borderline examples (e.g., bedtime‐in‐shelter planning is now coded as family‐level).

The integration of the photo‐elicitation method within this constructivist paradigm further enriched the analysis by emphasizing participants' voices and co‐constructing meaning through their visual artifacts. This combination of theory‐driven and iterative coding, supported by collaborative processes and credibility checks, ensured that the study's findings were both methodologically rigorous and deeply reflective of participants' lived experiences (Levitt et al., 2018) (see Table 2 for digital coping levels).

**Table 2:** Digital coping strategies across ecological levels.

Digital coping strategy & definition***	Personal level (self‑focused)	Family level (intra‑household)	Community level (multi‑household)
**Instrumental support‐** *Seeking advice, assistance, or information in the WPG on how to deal with the situation.*	Parent asks “Is there talk of escalation?” to decide whether to move their own children to the shelter.*	Parents post a checklist in the family chat—each child packs their labeled “grab bag,” and everyone practices reaching the shelter within 30 s.**	Several households coordinate a shared evacuation convoy and divide tasks (driving, supplies, lodging).*
**Planning‐** Using the information in the WPG to *think about‬ what steps to take and how best to handle the problem.‬‬‬‬‬‬‬‬‬‬*	Parent asks for school‑schedule updates to plan work‑day logistics.*	Parents check homework guidelines in the WPG, then brief all siblings together.**	Members crowd‑source real‑time road‑closure maps so the whole neighborhood can choose safe routes.*
**Emotional support‐** *Obtaining moral support, sympathy, or understanding from the WPG.*	Parent receives a single “We're with you 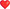 ” message that eases their anxiety.**	Spouse reads WPG empathy messages aloud to reassure younger children.**	Group launches a rolling “check‑in chain,” each family posting safety status and words of comfort.**
**Humor‐** *Making fun of the situation in the WPG.*	Individual posts a meme or one‑liner to lift their own mood.**	Family shares siren‐impersonation GIFs in their WPG to lighten the mood.**	Cascading riff in the WPG where several members build on each other's jokes, creating shared levity.*
**Venting‐** *Expressing negative feelings in the WPG.*	Parent types “I can't take another alert!” to release tension.**	Couple discusses fears privately, then one parent uploads a summary rant to the WPG.**	Multiple parents post successive complaints about municipal response, sparking a collective push for action.*
**Behavioral disengagement‐** *Disengaging from communication in the WPG.*	Parent mutes the WPG during sirens to lower personal stress.*	Household agrees to set phones aside in the shelter so children aren't left alone.**	Several members temporarily exit the WPG, prompting a meta‑discussion about information overload.*

* These coping strategies stemmed from the data.

** These coping strategies stemmed from the literature.

*** Definitions are based on Carver et al., 1989.

### Researcher reflexivity

As researchers, we approached this study from a constructivist perspective, recognizing that social realities are co‐constructed through human interaction and shaped by cultural narratives. Our positionality as researchers was influenced by our cultural and professional backgrounds, as well as our proximity to the Israel‐Gaza border. The current cycle of horrific violence involving all parties has and will continue to have major consequences for many people in the region for years to come. Despite these challenges, researchers in Israel, both Jewish and Arab, continue to engage in joint efforts to promote dialog, cooperation, and ultimately, peace. One of the authors specializes in digital coping, another focuses on resilience in conflict zones, and the third has expertise in mental health outcomes across diverse populations. These perspectives informed our interpretation of how digital tools are utilized as coping resources, and our interdisciplinary approach enabled us to critically reflect on our biases. This process ensured that the narratives we are presenting are firmly grounded in participants' lived experiences rather than shaped preconceived assumptions.

## FINDINGS

The WPGs functioned against a backdrop in which the specter of violence and the need for immediate, reliable communication were parts of everyday life. Within this milieu, participants found themselves grappling with the immediacy of threats and the perennial need to make rapid decisions impacting the safety and psychological well‐being of their families. The WPGs were found in this study to be a valuable resource for parents coping with political violence, providing emotional and instrumental support and fostering resilience and community cohesion. However, the focus on parent groups, rather than family groups, resulted in less emphasis on family coping strategies. Our findings are presented at the personal, family, and community levels, including quotes from interviews and anonymized and translated screenshots.

### Personal level

Our participants noted that the WPGs served as a personal coping resource for them to actively cope with the stress, by enabling the exchange of instrumental and emotional support and allowing participants to vent. Nevertheless, a few participants mentioned the anxiety from information overload and preferred to cope with emergencies through disengaging from group communication.

#### Instrumental support

Twelve participants described the local WPG as an instrumental coping resource used to seek advice, assistance, or information during missile attacks. The group provided updates on missile hit locations and ambulances, helping to alleviate stress and organize practical thoughts. One coping strategy was planning emergency responses, such as deciding whether to put children to bed in bomb shelters, on the basis of WPG information. Rachel said:

The group helps you decide whether you should have the children sleep in the bomb shelter tonight. If you know in advance from the group that there is a security flare‐up and missile attacks, then you don't want to wake up the kids in the middle of the night and run with them to the shelter.

**Figure 1:**
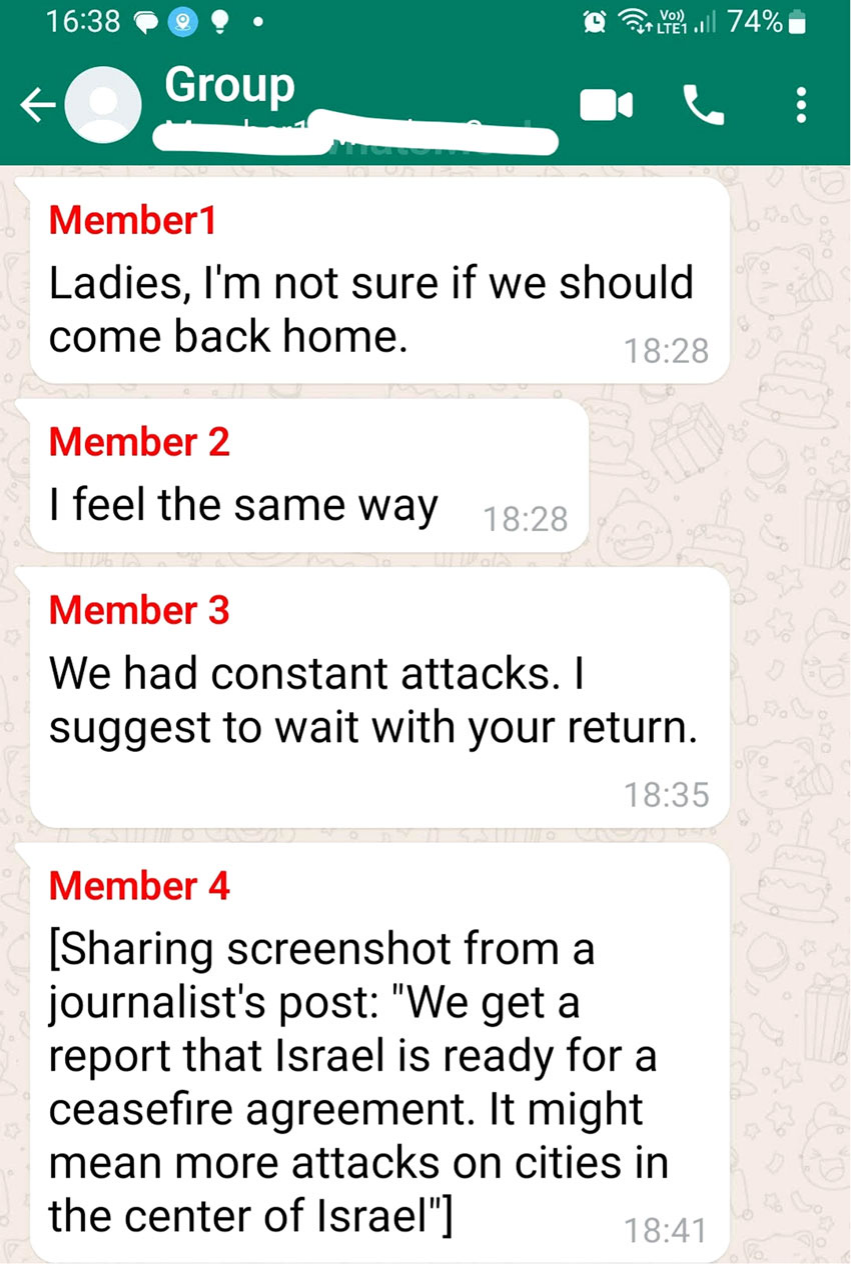
Searching for instrumental support in the group.

For many participants, communications in the WPG helped them decide during escalations when it was the right time to evacuate their homes, as Yafit said: “There is a lot of the following kind of discourse: *Are you evacuating? Did you start packing? We are debating*. It's a discourse that helps you understand where you are in relation to the others.” The WPG also served as a place to consider when the right time was to go back home after evacuating. For example, Inbal shared a screenshot (Figure 1) in which one of the group members, who had left town, asked for members' advice about coming back home.

As seen in the quotes above, the WPG served the parents as an instrumental coping resource. They used it to receive trustworthy information, to plan responses in the event of emergency, and to help with decision‐making regarding emergent conflict‐related issues such as evacuation and returning home during times of violence escalation.

#### Emotional support

In addition to serving as a personal resource for instrumental support, eight participants (out of 21) noted that the local WPG served as a resource for emotional support: obtaining moral support, sympathy, or understanding from the group members. Those who reported the need for emotional support were, in particular, mothers who had been left alone with the children. All Israeli men under the age of 40 who complete their compulsory service in the Israel Defense Forces from ages 18 to 21 are called up for reserve duty to provide reinforcements during emergencies (war, military operations, or natural disasters). Hence, during escalation periods, mothers are often left alone with their children to cope with missile attacks and other conflict‐related events. They remain constantly vigilant, anticipating the possibility of rockets, incendiary balloons, and terrorist infiltrations. These infiltrations are a persistent concern, as the Palestinian militant group Hamas repeatedly attempts to cross the border, whether through the border itself or via cross‐border tunnels (Richemond‐Barak & Voiculescu‐Holvad, 2023; Slesinger, 2020). Thus, emotional support was essential for this group of women, as Amit said:

As a mother who is often in an emergency situation alone, as a “single mother” of children, the group is another responsible adult standing there alongside me. It's a feeling that not just one person, but another 50 people, are with me, who are standing by my side and can give me advice whenever I need it. When you are in an emergency and feel alone, you are not in a good position; you don't function, which is a terrible feeling.

Within this framework, the additional stressors faced by those who are at home during escalations—often mothers with children—provide evidence of the civilian cost of conflict. The information shared in WPGs often bears the weight of this reality, providing not just updates on safety measures but also emotional lifelines for individuals isolated by circumstances. For example, Natalie shared such a moment, watching a helicopter operation taking place through her living room window and fearing a terrorist infiltration:

My husband was at home, sleeping on the couch. I remember waking him up: “Yuri, wake up, something is happening!” He wakes up and says: “What, what do you want?” He failed to understand the pressure and my distress. I turned off the lights and locked the house, the logical thing to do at that moment. He didn't understand what I wanted from him, and he turned the light back on [laughs]. At that moment, I wrote a message to the group, and the members immediately understood how I felt.

#### Venting

In addition to emotional support on the personal level, the group also provided our participants a space to vent their negative feelings and emotions. At the individual level, venting in WPGs involves parents expressing their personal frustrations, fears, or emotions in response to stressful situations. These expressions are typically self‐focused and serve as an emotional outlet for managing personal distress, with responses from the group being secondary, as Ye'ela said: “You feel like you can vent: ‘Wow, that boom was intense.’ Someone else might have heard something too. There is something helpful about it that we're all in it together.”

**Figure 2:**
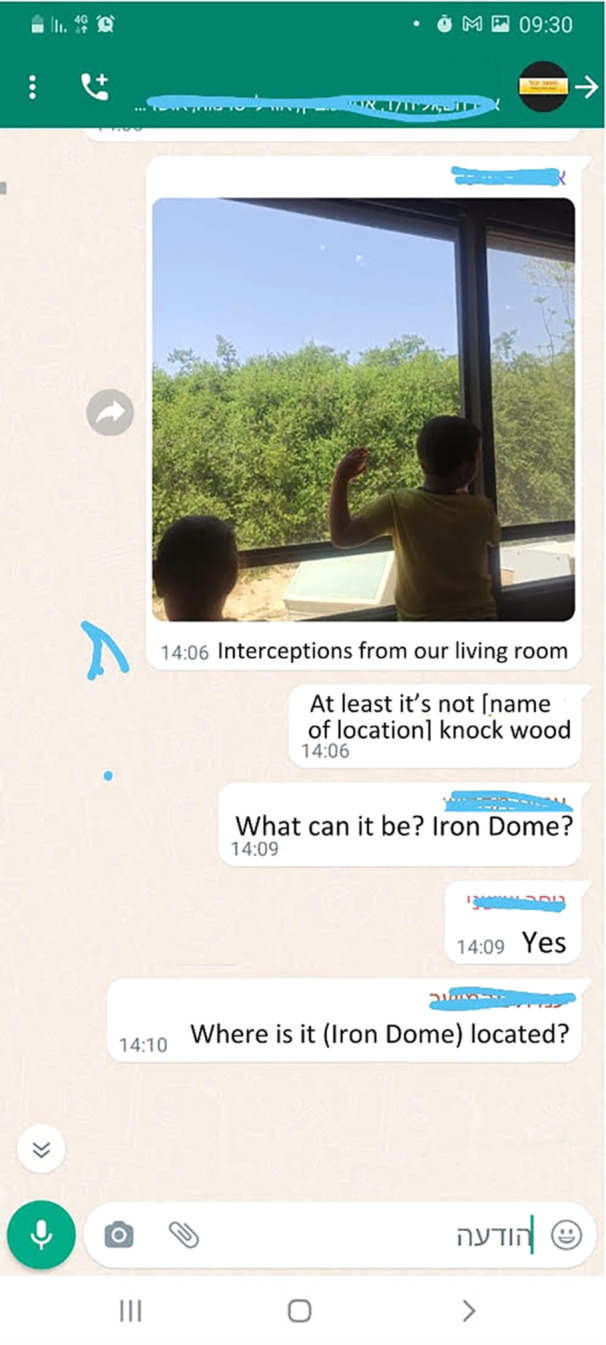
Unsupportive communication in the WPG. WPG, WhatsApp parent groups.

The constant flux of information, some of it urgent and lifesaving, some of it distressing, and some misleading, reflects a larger picture of a community under threat. The technological threads that bind them are the same ones that can unravel their sense of security, leaving them with a complex tapestry of community, isolation, information, and misinformation. Orit shared a screenshot (Figure 2) from the group in which one member sent a photo of his kids watching the Iron Dome (Israeli air defense) missiles intercepting the Hamas missiles. In addition, another member shared the exact location of this incident. Orit described this group interaction as stressful and defeatist: “Getting these interception pictures heightens my anxiety. I don't feel they send a supportive or reassuring message. On the contrary, I'm angry about the lack of awareness of sharing strategic locations of rocket hits, which hostile sources can trace.”

**Figure 3:**
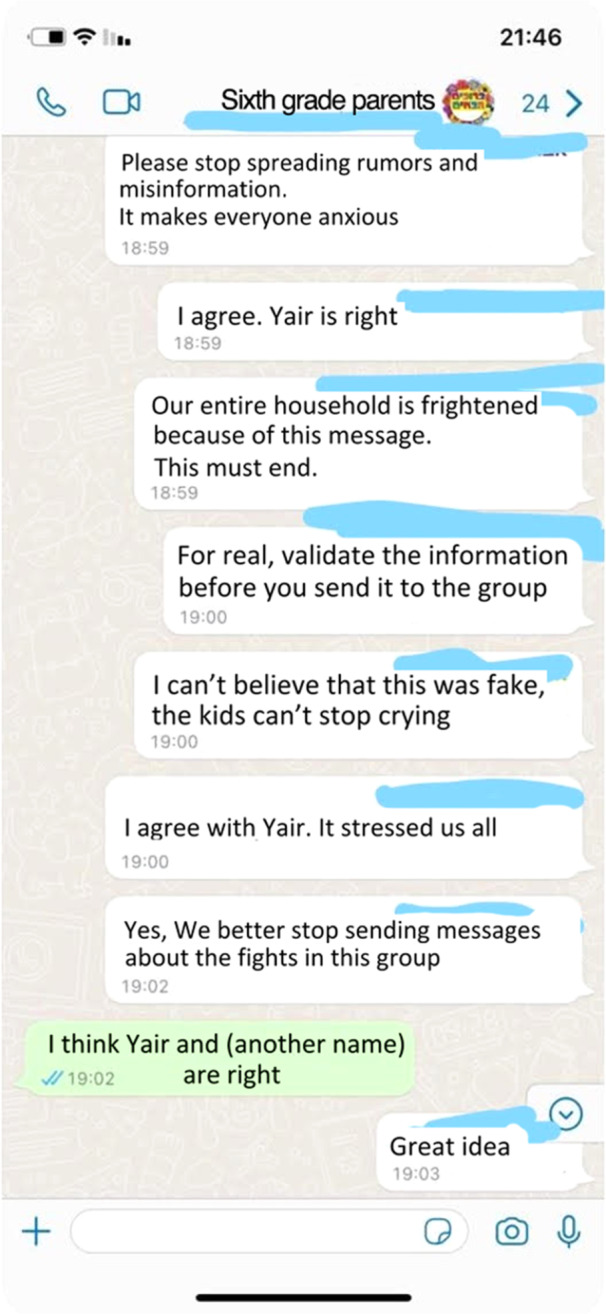
Response to misinformation spread through the group.

In addition, some group members worried that their communication might distress others. For example, Natalie felt that her anxious message may have caused others to feel stressed: “I'm afraid I may have made other women stressed as I don't know what state they were in.” Moreover, as described next, in some cases the overwhelming emotions and information in the group led members to disengage from group communication (Figure 3).

In some cases, the WPGs were prone to receiving and spreading misinformation—that is, false information regardless of the intent to mislead. Although the proportion of misinformation relative to other types of information exchanged in WPGs was relatively small, it was nonetheless impactful. During emergency situations, such misinformation—false messages about casualties and ongoing threats—hindered coping efforts. Orit described the fear elicited by a false message, recalling an incident where, after a rocket hit the kibbutz, “someone wrote there might be an injured person, even though there wasn't.” Diana shared a screenshot (see Figure 3) of such an incident, to which group members responded fiercely:

In the last escalation, while we all sat in the shelters, someone spread a rumor that a rocket had hit the children's school. It was a nightmare. It was forwarded between neighbors, and there was a discussion about it in the group. My screenshot is of when one parent wrote angrily not to pass on unverified information: “Do not report something that did not happen. It stresses everyone out.”

Irit shared another case of misinformation, during which a group member sent a message warning about a terrorist infiltration in her town:

Believe me, you don't want to imagine what a mess it created. We were all stressed. It was a terrible fear, for all of us, including me, my husband, all the adults, and the children. If there is a terrorist in town, he could enter my house and kill everyone. It's terribly, terribly scary to get such a message.

Despite the advantages WPGs offer, they also expose the vulnerability of participants to the detrimental effects of rumor‐mongering, which can lead to panic during already tense times. This situation underscores the delicate balance of being informed and being overwhelmed, where too much information can paradoxically lead to a paralysis of action or exacerbate the fear and anxiety it seeks to mitigate.

#### Behavioral disengagement

As previously mentioned, the WPG inadvertently became a source of distress for some participants. Consequently, disengaging from the group's communication and ignoring messages on WhatsApp emerged as a coping strategy. This approach was exemplified by Gila's experience:

I prefer not to be constantly bombarded by updates, especially during emergencies. I don't need to know every detail of where each rocket fell. While I understand that some people may want this level of information, it's not helpful to me. I choose not to be in groups where this is the focus.

Interestingly, Inbal described herself as being better off *avoiding* information that was coming from the WPG: “I decided not to read messages in the group during days of missile attacks. This is *my way* of being resilient—by controlling what information I want to be exposed to.” Hence, what started as a valuable and ubiquitous source of instrumental and emotional support became another source of distress for some. Yaron, one of the participants who left his local WPG, stated that the unceasing communication in the group during emergencies led him to leave the group:

In the last escalation, my wife said, “Let's put the phones away. If there is a missile alert, we will protect ourselves and not depend on the phone.” And that's what we did, and it relieved the anxiety. I think, in this sense, the WhatsApp group only raises the level of anxiety just like when the TV and radio are on. It surrounds and overwhelms you.

The continuous threat of rocket attacks and the psychological toll of living with such unpredictability require coping mechanisms that go beyond traditional support systems. WhatsApp parent groups, in this context, serve on the personal level a dual function of crisis management and social support.

### Family level

Based on our participants' responses, it seems that the use of WPGs did not significantly contribute to family coping strategies when dealing with stressful situations. Although parents often relayed information from WPGs to their families, these instances were classified as individual‐level coping because the parent acted as an intermediary rather than engaging in collective family discussions or actions. As noted in the previous section, one parent described how they gathered safety information from the WPG to decide independently how to prepare their children for emergencies. Although these decisions impacted the family, they did not involve joint problem‐solving or collaborative coping, which were required criteria for family‐level impacts. We identified only one case in which a family used the local WPG as an informational coping resource.

#### Informational support

The May 2021 conflict between Israel and Gaza involved rocket attacks on Israel by Hamas and Islamic Jihad, Israeli airstrikes in the Gaza Strip, as well as protests and police riot control inside Israel, leading to road blockages. Orit shared how they utilized a local WPG to plan their hurried evacuation to a safe place:

A friend shared a photo in the group of a car that was stoned next to the village of Rahat [a nearby Bedouin town]. This road is on our usual route to the highway up North. In this case, the group information was relevant to us because we planned our evacuation route on the basis of such messages to avoid as much as possible arriving at the scene of a riot…So, it helped us plan our trip when we had to flee.

In this example, informational support is categorized as a family‐level resource because the shared information in the WPG was used to coordinate a family‐level decision and action (i.e., ensuring the safety of all family members during the emergency). Although an individual player may have accessed and used this information personally, its impact was directed at enhancing collective family coping— namely, determining an evacuation plan for the whole family. As such, we found this resource to be distinct from purely individual coping resources, which are typically limited to strategies employed for the personal benefit of the individual engaging with the WPG.

#### Behavioral disengagement

Most of our participants reported that they sometimes intentionally refrained from using the local WPG to manage their families during emergencies. Gila explained that the group communications undermined her family's coping efforts: “It's between me, my husband, and the kids. I don't really care what others do. If my coping mechanism is to stick to our usual routine, then reading that others are responding differently can be destabilizing.” Ruby described his family's disengagement from the WPG when coping with stressful situations: “During missile attacks, we run with the kids to the shelter. If we run to our phones, then the children are left alone to deal with the noise and everything else.”

Behavioral disengagement, such as avoiding the WPGs in favor of direct action (taking children to shelters), points to a prioritization of immediate physical safety over the potential benefits of communal information sharing. This act of refraining from digital communication during an emergency is a calculated decision to focus on direct action, which can be critical for family unity and children's sense of security during such events. Although WPGs can be a lifeline for some, providing timely updates and a sense of community support, they can also be a source of additional anxiety for others, who may choose to opt out, to maintain psychological equilibrium within the family.

### Community level

At the community level—defined here as WPG interactions that mobilize multiple households for the benefit of the wider community rather than a single family—our participants' accounts illustrate how local WPGs can aid them in collectively acting and trying to improve the already complicated situation in which they live during emergencies. Through communications in the WPGs, they can concentrate on doing something together to protect their community. Our analysis revealed four main forms of digital coping: instrumental support, emotional support, humor, and venting. Nevertheless, in some cases, the local WPGs can serve as a venue for the collective expression of anger and frustration toward local organizations or become a source of distress.

#### Instrumental support

In addition to their active coping with emergencies, the WPG served as a significant source (for participants) of collective instrumental support. Thirteen participants noted that community members used it to obtain tangible help and advice. One form of support was organizing community action to support injured fellow residents or those whose homes were damaged during missile attacks. Rachel said, “Many were joining in to help those in need with donations of groceries and other means.” In one case, a community made use of the local WPG to support a bereaved family whose 5‐year‐old child was killed when sheltering with his family in the bomb shelter (i.e., a piece of shrapnel managed to land in the shelter itself). Rachel said: “In this specific case, the groups were very active and organized to help during the shivah [period of mourning], with food and visits. It was very moving to see and be a part of it.”

During escalations, some group members used the WPG to organize recreational activities or playdates in safe spaces for the children. For example, Esther's family, who lived in a longstanding locality where many old houses lack bomb shelters, described how the WPG group members used it for this purpose:

In the kindergarten [WhatsApp] groups, parents invite other families to come and pass the time in their bomb shelter, or provide help if they need help with something else. There is a lot of support for parents in keeping the children safe and active together in the group. For parents, it's a relief to bring them together. It makes it easier for them.

One unique use of the WPGs as a communal resource in an ongoing conflict is for the informing, planning, and organizing of local evacuations during emergencies. Natalie used the WPG to organize a communal evacuation to a town outside the missile range. She posted a message in the group asking interested members to join and described how she used the WPG to plan and execute the evacuation:

I got there at 6:30 am. I saw the apartments, called the other members, and told them what was there, what was missing, and what the apartments looked like. I took a video for them. Afterward, we sent more messages through the mothers' group inviting more mothers. In the end, there were eight families there.

The local WPGs were used for coordinating local evacuations, offering information on safe spaces, and even helping to organize where families could stay outside the danger zone. As the quotes above describe, our participants used the local WPG to collectively care for members' families while under fire.

#### Emotional support

**Figure 4:**
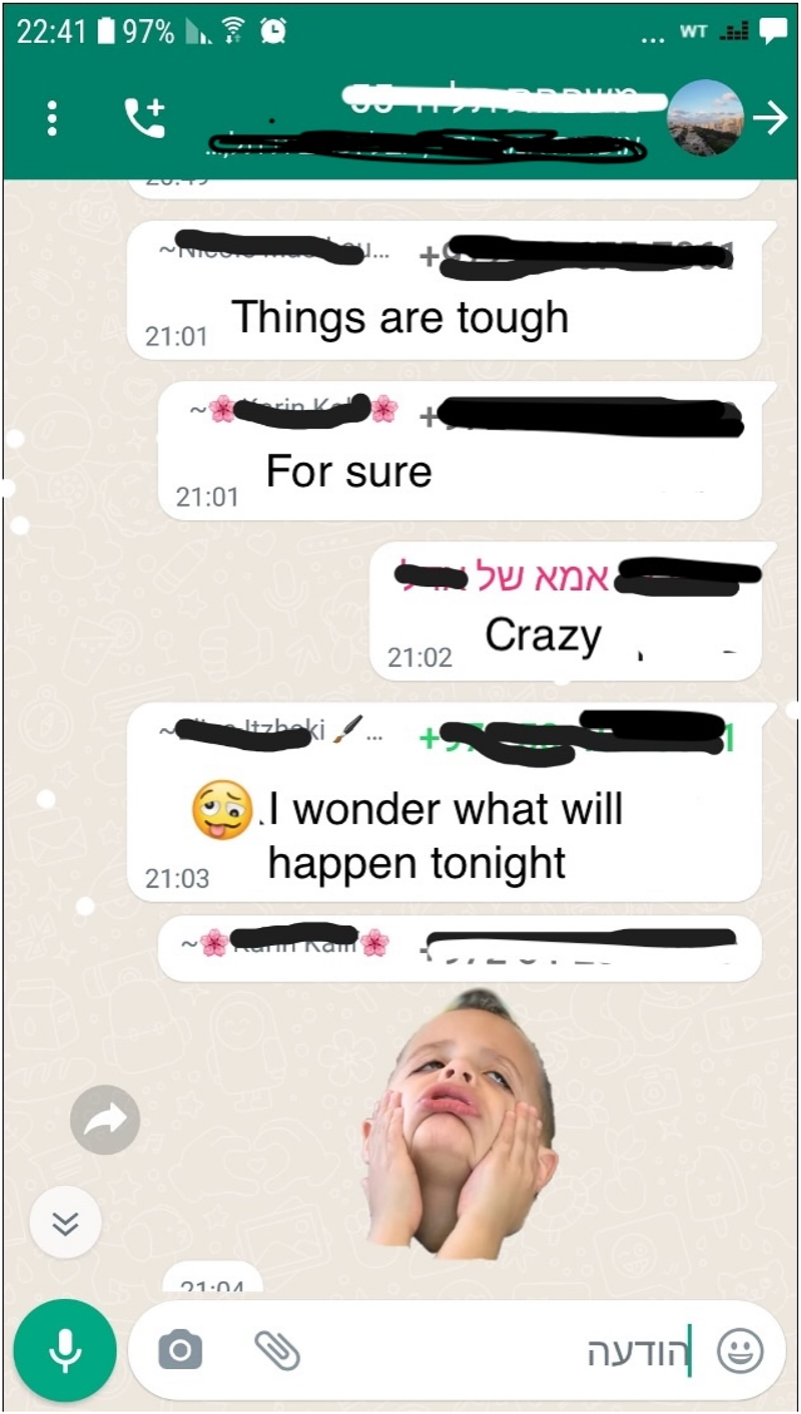
Emotional support exchange as a communal resource.

The WPGs also acted as platforms for emotional support, where community members could express their fears and seek comfort (see Figure 4). Personal outreach was also facilitated through WPGs, with members providing direct support to those who were visibly struggling. Ella shared a screenshot of a member's request for help for her child: “She wrote that her child was shaking and hadn't slept all night. The group members expressed empathy. They provided a sense of identification that we were all in the same boat.” In many cases, the request for emotional support in the group led some members to reach out directly to the member in need. For example, Natalie said, “We know how to identify it when someone is in need, and then we approach her. We say, ‘Listen, we can tell that something is going on with you. You should go talk to someone at the local [social] services.’” Unlike other WhatsApp groups, which can overload members with unnecessary information, for some participants, the WPG serves as an essential digital group for emotional support, as Ye'ela noted:

We never put the group on silent mode. There is no unnecessary quarreling in the group. I remember one night when there was an emergency. We were up texting all night. After someone wrote that she'd had an anxiety attack, I went over to her house and helped her.

The quotes above illustrate how group members collectively coped with stressful situations by obtaining comfort and understanding from other group members, and often as a platform to coordinate response efforts and aid provision. For many members, the WPG became a significant source of ongoing emotional support where they felt included and understood.

#### Humor

**Figure 5:**
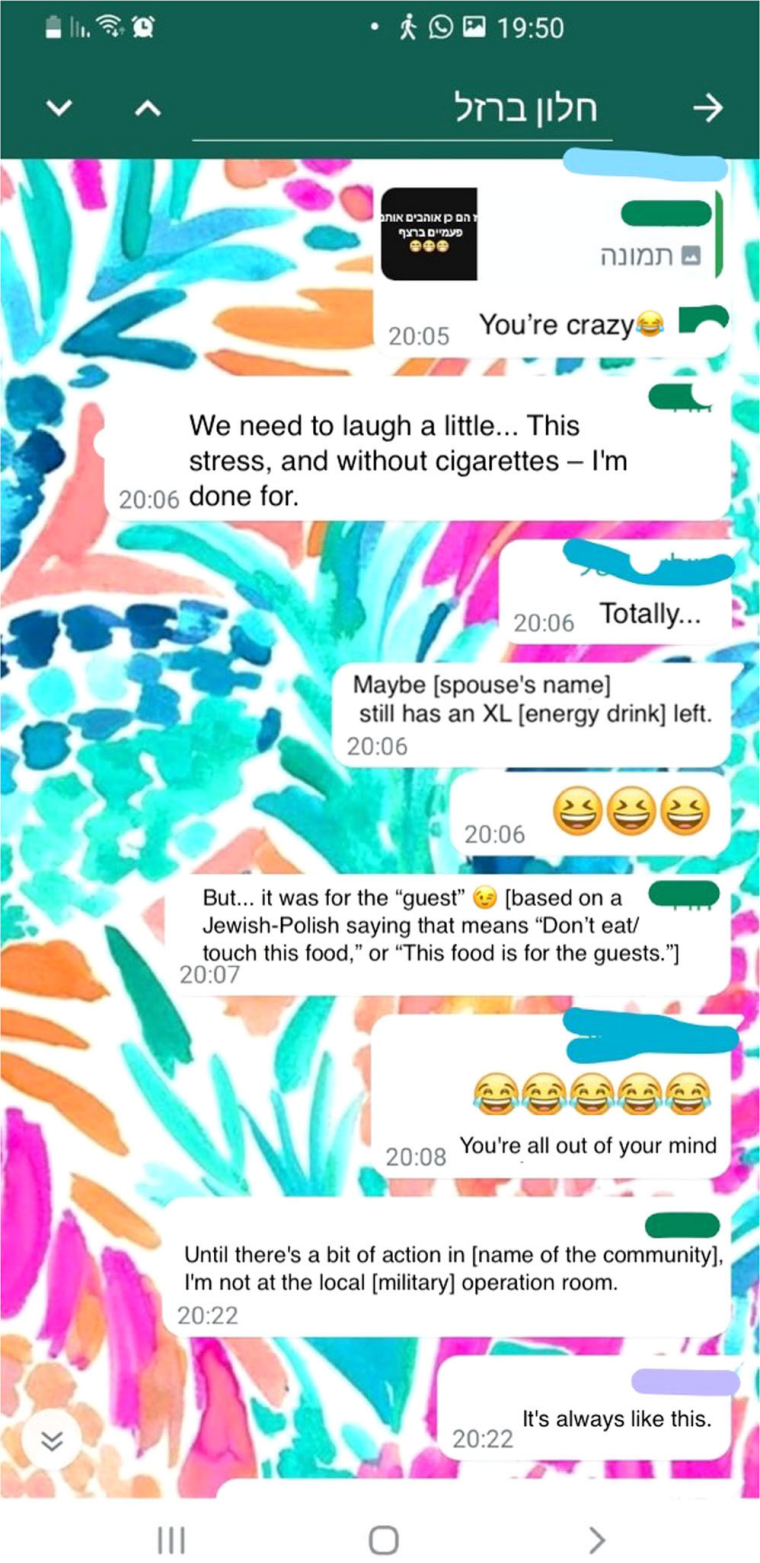
Humor as a digital communal resource.

One interesting digital coping strategy participants mentioned was making fun of the emergency situation in the WPGs. Participants would send jokes about the emergency, share funny memes from local Facebook groups, and make references to children's books as a way to lighten up the situation. For example, Natalie shared a screen shot (see Figure 5) of interactions in the local WPG during a 6‐h lockdown in her local municipality. Military officials had been concerned about a terrorist infiltrating the municipality and had ordered residents to stay at home, lock the doors, and dim the lights. In the WPG interaction, one of the members shared a funny meme, and another member responded, “You're nuts.” The first member replied, “We've got to laugh. Just a little—all of this stress and no cigarettes left—I'm a wreck.” The other members responded with laughing emojis and agreement.

In another case, Amit shared a post from a humorous Facebook group in which the names of the Israeli villages bombarded along the border were compared to an attendance list of a pre‐school. Amit explained: “Black humor often appears in our group. That's our way of dealing with the situation. Despite the tough reality, we can still laugh together and make fun of it.”

#### Venting

Besides humor, some members noted that venting and expressing negative feelings in the WPG was another collective digital coping strategy used by the group. In contrast to the personal level, venting at the community level involved expressions of shared frustrations or concerns that represented collective experiences. This type of venting often highlights systemic issues, such as dissatisfaction with government responses, and serves as a platform for group dialog and solidarity. For example, parents might collectively voice frustration about inadequate municipal emergency responses, using the WPG as a forum to exchange ideas or propose collective actions. Yafit served as the kindergarten coordinator in her locality and notified parents about the county's decision to temporarily close the school as a precautionary step. As a result of that announcement, some parents felt frustrated and vented their frustration on the local WPG:

One mother wrote [in an aggressive and nervous manner], “What? They're closing again?????? And why? This isn't what the [government] instructed!” The whole conversation goes in this direction, igniting the entire community. It might lead them to contact me. I will try to show understanding and pass the message on, but I can do nothing about it. The group becomes this place that produces negative discourse. And people who never thought they would be in such a place of hostility, or of blurting out unpleasant things, now have a place to go where it's easy to insult and bad‐mouth others.

Our participants also used their local WPG for a dialog about the children's school's response to the killing of a schoolmate as a result of a rocket attack. Following this tragic incident, and as the school was closed, the school administration sent a message stating that the children were invited to join a class Zoom meeting with their homeroom teacher and a psychologist to talk and share their feelings following the loss of their schoolmate. Alongside the posting of this message in the group, a discussion arose within the group about whether or not it was appropriate for the children to participate in the school's initiative. Gila shared with us a screenshot of parents' responses expressing their thoughts on her decision not to allow her children to join the Zoom meeting. In the group, participants thanked her for sharing her reasoning for not having her children attend the meeting. Gila said:

There was a relevant and thoughtful parental discussion in the group about a very complex and challenging issue. You can also see that there weren't many responses. It was only me, the person who posted it, and maybe two others who responded. I am sure there were other parents who read it and found themselves somewhere on the spectrum of these perspectives.

In some cases, the expression of negative emotions in the WPGs led some participants to ignore the conversations and even leave them. For example, David noted:

The group is the opposite of a source of support. For example, there are often many comments where parents express their concerns. I hardly ever read it because I put the group on silent. But when I do see it, it's often anxious messages.

Despite the occasional negative effects, the WPGs play a multifaceted role in these communities during emergencies. They act as a means of coordination for practical support, a source of emotional relief, a platform for shared humor, and occasionally a place for the venting of frustration. The quotes provided illustrate the deep reliance on and value of these digital communities in times of crisis.

## DISCUSSION

The aim of the current study was to explore the role of local WPGs as digital coping resources from the perspective of three ecological levels—individual, family, and community—in the Israeli communities bordering Gaza, one of the most volatile regions in Israel. Our analysis revealed that participants actively adapt WhatsApp features to address the challenges of ongoing political violence at two ecological levels: personal and community. At the individual level, these strategies are self‐focused and often emotionally driven, whereas community‐level coping is collective in nature and often aimed at mobilizing group discussion or actions. These strategies reflect parents' agency in using technology to meet their immediate needs, reflecting broader cultural narratives and social practices while navigating the challenges of their environment.

In contrast to our expectations, study participants did not describe digital coping activities at the family level, referring to the extent to which individuals use the WPG as a family resource to strengthen the family unit and reduce the impact of stressful life events (Bronfenbrenner, 1986; Bronfenbrenner, 1992). Although the WPGs provided valuable information and emotional support, much of the parents' engagement occurred at the individual level, where they independently decided how to act on the information they received. These findings may stem from our focus on WPGs rather than on family WhatsApp groups. If we had studied digital coping in WhatsApp family groups, we may have been able to identify family‐level digital coping, as parents don't see their community WPG as a space to discuss private family issues or coping activities.

At the personal level, our study indicates the ways parents identify the external resources (i.e., local WPGs) at their disposal and use them to promote individual coping efforts (Eriksson, 2017). This finding reflects Lupton's (2022, 2023) social construction of technology framework, as parents' use of WPGs demonstrates how digital tools are socially constructed through their practices and needs, turning WhatsApp into a personalized coping resource during crises. However, despite the benefits, some participants felt overwhelmed by the information in these WPGs and chose to disengage from group communication. Our analysis revealed that exposure to WPG communication may have impeded members' coping efforts, especially in the event of misinformation or rumors that ran counter to efforts to feel safe and informed. Participants emphasized the emotional toll of dealing with unverified information, reinforcing the need for group protocols to minimize the spread of harmful or misleading content. In addition, being part of a local WPG can, in and of itself, become a stressor when the expression of negative feelings by others becomes the dominant discourse in the group.

On a community level, our study indicates that local WPGs can be helpful in addressing emergencies collectively through the provision of instrumental support, emotional support, relief through humor, and as a platform for venting. The ways in which participants adapt WPGs for communal coping underscore the socially constructed nature of these digital technologies (Lupton, 2022, 2023). These groups are not just platforms but are co‐created spaces where parents negotiate the boundaries and functions of technology in addressing collective challenges. Participants’ narratives also illustrated how families and communities adjacent to the Israel‐Gaza border faced heightened stress and trauma due to the unpredictability of rocket attacks and the need to remain hypervigilant (Levi‐Belz et al., 2025). In some cases, the stress was amplified by structural inequalities, such as reliance on community resources when formal services fell short.

In some cases, the WPG forum can become a source of frustration and anger toward local organizations. Participants expressed frustration when local organizations could not meet their expectations, reflecting a broader discontent with institutional support. Moreover, our results indicate that behavioral disengagement and venting represent complex dynamics within WPGs, where venting serves as both a coping mechanism and a potential stressor. For example, some participants in our study expressed feeling overwhelmed by the negative tone of others' venting, which at times led to their disengagement from group communication. This finding aligns with findings from other studies on online communities, which demonstrate that excessive negativity can reduce group cohesion and discourage participation (Aakhus & Rumsey, 2010). Additionally, research on digital social support in contexts such as online forums shows that unmoderated venting can alienate group members, particularly when it fosters conflict or amplifies stress rather than providing relief (Fullwood, 2016; Oladeji et al., 2024). Therefore, moderation is a critical factor in sustaining the functionality of online groups such as WPGs. The presence of moderators who actively maintain group norms, address conflicts, and manage the flow of information can significantly enhance group dynamics and ensure that venting remains constructive (Figueras Bates, 2024). Overall, despite the abovementioned challenges, WPGs significantly enhanced community preparedness, fostered social trust, and strengthened collective efficacy, contributing to community resilience (Braun‐Lewensohn & Sagy, 2014).

The study's significance lies in the identification of types of social support and coping strategies present at both personal and communal levels within the digital landscape. Previous research on coping has primarily examined ecological coping strategies and their interplay in offline settings, overlooking digital media's role as a potential coping resource. In this study, the WPGs functioned as micro‐level systems within a larger ecological framework. For example, parents assumed dynamic roles, such as information disseminators or coordinators during emergencies, interacting with external systems such as schools, local governments, and the military infrastructure. These interdependencies reinforce the importance of considering how WPGs operate as localized systems embedded within broader community and macrosystemic structures. As such, the current study contributes empirically to our understanding of how individual and collective coping strategies are utilized on digital platforms by parents in conflict‐ridden realities.

Further, we explored how digital coping mechanisms and community perceptions could be instrumental in understanding social media's role in meeting the social needs of those living in conflict zones. We investigated how being part of such online communities could enhance people's sense of community, safety, and personal well‐being. These insights are vital for residents, professionals, and formal support group facilitators in conflict‐ridden areas to develop communal strategies that bolster personal and communal resilience.

Our findings exemplify the social support functions of digital communities, especially in contexts where traditional forms of social and governmental structures are under strain. Within this context, WPGs function as critical tools for coping and communication, but their effectiveness is shaped by these larger structural realities. For example, although WPGs provide immediate emotional and instrumental support, they also reflect the heightened anxieties and misinformation that can circulate in a community under duress. These dynamics highlight the interplay between the macrosystemic influences and the micro‐level coping mechanisms explored in this study.

As participants navigate the ebbs and flows of conflict, the local online communities stand as digital monuments to resilience, adaptability, and the human capacity to find solidarity amidst chaos. They reflect a microcosm where modern communication meets the need for belongingness and a sense of protection, revealing the intricate dance of coping mechanisms that are employed when facing the realities of conflict—a dynamic co‐construction of technology, with the platform continually redefined to meet emotional, informational, and community‐level needs. Given that these groups serve as a hub for diverse social support mechanisms and can alleviate the psychological toll on individuals and communities, grasping the function of digital groups can inform approaches to bolster community resilience amidst the pressures of life in conflict‐ridden areas.

Conversely, our findings also demonstrate how social interaction involving misinformation and triggering messages in digital settings can harm personal and communal coping efforts. Local online communities may also interfere with personal and collective coping efforts. This interference happens when threats become too frequent, and users feel overwhelmed by the amount of chatter in the groups; when people lose trust in local institutions' instructions and use online communities to express their frustration; and when rumors about casualties and damage spread on local online communities.

The knowledge gained from this study can assist policymakers in creating supportive infrastructures that leverage technological solutions for communal assistance, leading to improved measures designed to aid residents in conflict zones. We advocate for policymakers to formally recognize local online communities within emergency plans to bolster communal support through social media. We urge policymakers to collaborate with local authorities and experts to develop a cohesive digital communication strategy. Developing protocols for emergency support that incorporate digital communication is crucial, as is establishing partnerships with mental health and media professionals for expert guidance on managing online communities. Local authorities should offer crisis communication training to local online community administrators and disseminate educational resources about the effective use of social media during crises. Such guidance should advocate for the promotion of humor and positivity and provide communication strategies to help alleviate panic. Guidance should also include tactics for handling negative discourse by instructing group administrators in conflict resolution and establishing protocols for constructive venting. Lastly, we recommend the implementation of systems to assess the effectiveness of local online communities and their impact on the physical community. By incorporating these recommendations, local social media can become an invaluable asset for communal support and resilience during emergencies, ensuring a harmonious balance between providing aid and preserving the well‐being of its members.

## STUDY LIMITATIONS AND FUTURE RESEARCH

There were four primary limitations of the current study. First, our participants were members of both rural and urban communities that varied in their social cohesion levels. For example, some communities may have had a strong sense of collectivism, whereas others may not have. Second, our sample may have slightly overrepresented urban residents and individuals with higher education and income levels compared to the predominantly rural and economically diverse population of the region (Israel Central Bureau of Statistics CBS, 2022). These differences should be acknowledged as a potential limitation when generalizing findings to the entire community.

Third, although this study emphasizes the proximal interactions within WPGs, Bronfenbrenner's ecological theory underscores the importance of broader community dynamics, including shared norms, interdependence, and resource cycling (Peirson et al., 2011). These factors highlight how communities, as larger ecological entities, provide critical structures for collective coping, beyond the immediate communal interactions observed within our sample. Future research could expand on these findings by examining how broader community structures, including institutional support and sociopolitical dynamics, shape and are shaped by digital coping mechanisms within conflict‐affected regions. Lastly, the stability of internet access and the availability of necessary resources in the context of our study support the ecological validity of our findings vis à vis the Israeli side of the Israel‐Gaza border. However, the external validity of our study may be limited when considering other conflict zones where internet access, electricity, and digital infrastructure are less reliable or even absent. For example, in Gaza and other such regions with power shortages (Netblocks, 2024), these constraints can significantly impact digital communication. Moreover, many countries worldwide block or filter internet content (Deibert et al., 2008), and in some cases, block platforms such as Facebook and WhatsApp (Digital Watch Observatory 2024). As a result, WhatsApp may not play as prominent a role in community and coping mechanisms in these contexts or may be adapted to fit such constraints.

Based on our findings, we suggest exploring qualitative research methodologies to delve into how parents manage stress within the family setting in areas typified by political conflicts, which might offer a deeper understanding of their coping strategies. Doing so could involve interviews, focus groups, or case studies that allow for a more nuanced exploration of family dynamics and the role of digital media in coping with stress. One such method might be using a photo‐elicitation technique during interviews with members of family digital groups. We also recommend developing a research tool dedicated to gauging digital coping based on ecological theory—that is, developing digital coping measures at the personal and community levels based on our attached interview protocol, especially when examining different levels of analysis. These insights may contribute to enhancing the effectiveness of online groups for the benefit of members and/or create new online platforms to support individuals facing political violence or other ongoing emergencies.

## CONFLICT OF INTEREST STATEMENT

The authors declare no conflicts of interest.

## Supporting information

Appendic A final.
