# Supplementary material for: Parents' WhatsApp coping resources in the context of ongoing political conflicts: An ecological exploration
Source: Am J Community Psychol. 2025 Sep 12;77(1-2):98–117. doi: 10.1002/ajcp.70017 (PMC13007763; doi:10.1002/ajcp.70017)
Supplement: Supplementary file 1 — Appendic A final. [file AJCP-77-98-s001.docx]

**Appendix A: Photo-Elicitation Interview Protocol**

Personal Coping Resources

1. Please share some basic details about yourself (age, marital status, and the number of years you've lived here).

2. Can you tell me when and why you joined the local WPG? Do you actively participate in group discussions? If so, why?

3. Discuss the screenshots you provided: Why did you select these particular interactions? What makes them meaningful to you? Can you give some context to these interactions?

4. How do you utilize the group in your day-to-day life? Can you provide some examples?

5. In emergency situations, how do you use the group? For instance, when an alarm sounds, what actions do you take on your phone? If possible, provide screenshots for clarity.

6. Which WhatsApp features do you typically rely on during emergencies (e.g., sending messages, sharing links, recording voice messages, sharing photos)? Again, if possible, please provide screenshots.

7. Conversely, which WhatsApp features do you avoid using during emergencies? Why is that?

8. Can you compare the support you personally receive from the local WPG to the support you get elsewhere? Please give an example. Screenshots would be helpful if available.

9. Are there any drawbacks to using the local WPG, in your opinion? Please explain with examples. Screenshots would be helpful if available.

10. What guidance would you offer to individuals living in areas with security concerns about using technological tools?

**Family Coping Resources**

1. Is your spouse part of any local WPG? What motivated your spouse’s decision to join or not join?

2. What kind of social support do you believe your family or partner seek in the local WPG? For example, are they seeking emotional support, informational help, self-esteem boosts, or practical assistance?

3. How do your family members or partner use the group during emergencies? For example, when an alarm sounds, what actions do they take on their phone?

4. Can you contrast the support your family receives within the group to the support received from other sources? Please provide an example.

5. In your opinion, what are the potential downsides of your family or spouse using the local WPG? If possible, if possible, please provide screenshots.

6. What advice would you have for families residing in areas with security concerns?

**Community Coping Resources**

1. Describe your relationship with your community. Do you engage in community events? Do you ever take the lead in organizing them?

2. How does being a part of the local WPG influence your sense of community belonging? Could you give an example?

3. In what ways does group membership aid the community's response during emergencies? Any specific instances you can share? Screenshots would be beneficial if available.

4. Has group membership altered the community's capacity to manage emergencies in any manner? Please elaborate with an example. Screenshots would be beneficial if available.

5. What do you perceive as the downsides of the community's use of the local WPG? Screenshots would be beneficial if available.

6. What recommendations would you give to community leaders in areas facing security challenges?
